# Supplementary figures and images for: Hortaea werneckii isolates exhibit different pathogenic potential in the invertebrate infection model Galleria mellonella
Source: Front Fungal Biol. 2022 Nov 29;3:941691. doi: 10.3389/ffunb.2022.941691 (PMC10512279; doi:10.3389/ffunb.2022.941691)

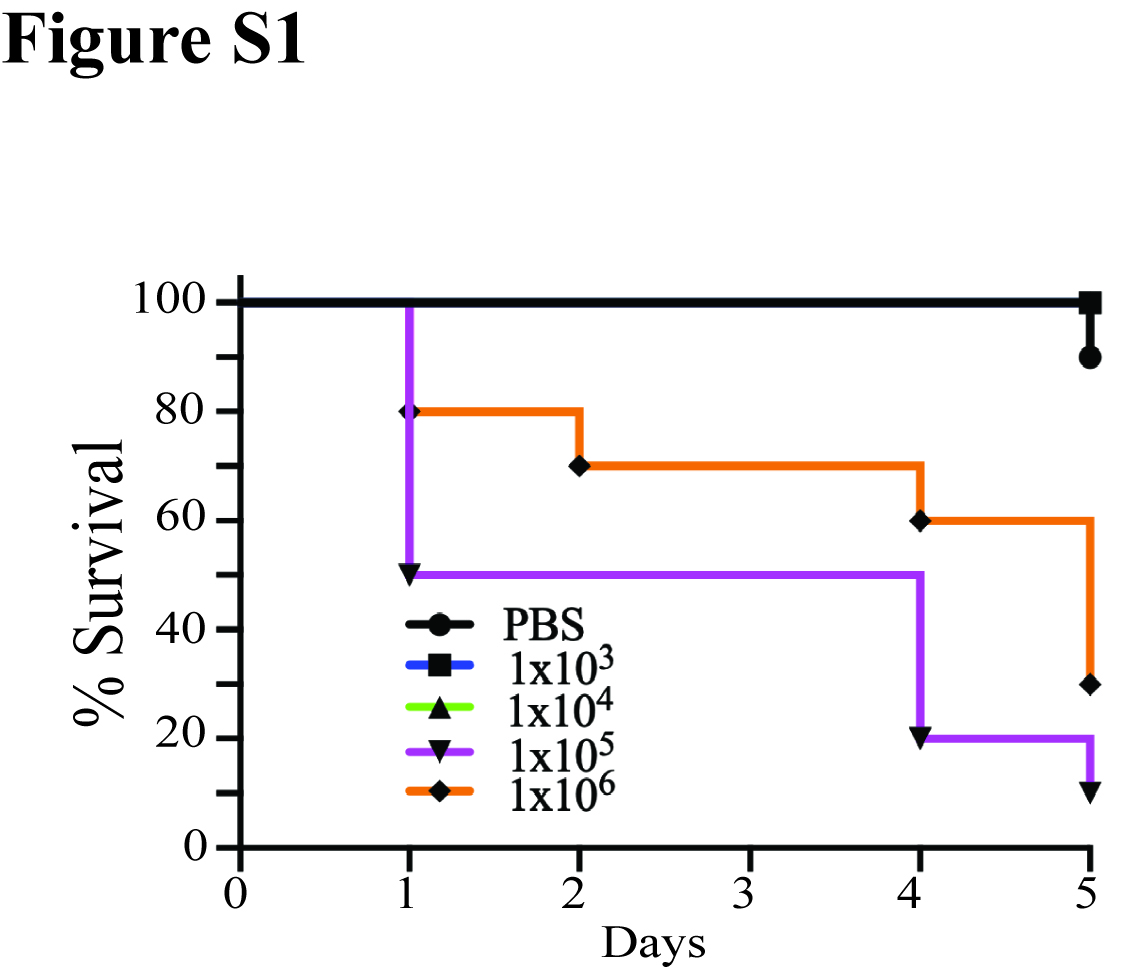

Supplement: Supplementary Figure 1 — Establishing an insect-based model to assess H. werneckii virulence. Different concentration of the EXF-2000 strain were injected into 10 Galleria mellonella larvae. Larvae were incubated at 37˚C and survival was monitored for 5 days. [file Image_1.jpeg]
